# How do barley plants with impaired photosynthetic light acclimation survive under high-light stress?

**DOI:** 10.1007/s00425-023-04227-8

**Published:** 2023-08-26

**Authors:** Monireh Saeid Nia, Louis Scholz, Adriana Garibay-Hernández, Hans-Peter Mock, Urska Repnik, Jennifer Selinski, Karin Krupinska, Wolfgang Bilger

**Affiliations:** 1 Institute of Botany, Christian-Albrechts-University, Kiel, Germany; 2 Leibniz Institute for Plant Genetics and Crop Plant Research, Gatersleben, Seeland, Germany; 3 Central Microscopy, Department of Biology, Christian-Albrechts-University, Kiel, Germany; 4 Present Address: Molecular Biotechnology and Systems Biology, TU Kaiserslautern, Paul-Ehrlich Straße 23, 67663 Kaiserslautern, Germany

**Keywords:** Excess excitation energy, Lutonarin, NPQ, Tocopherols, WHIRLY1, Zeaxanthin

## Abstract

**Main Conclusion:**

WHIRLY1 deficient barley plants surviving growth at high irradiance displayed increased non-radiative energy dissipation, enhanced contents of zeaxanthin and the flavonoid lutonarin, but no changes in α-tocopherol nor glutathione.

**Abstract:**

Plants are able to acclimate to environmental conditions to optimize their functions. With the exception of obligate shade plants, they can adjust their photosynthetic apparatus and the morphology and anatomy of their leaves to irradiance. Barley (*Hordeum vulgare* L., cv. Golden Promise) plants with reduced abundance of the protein WHIRLY1 were recently shown to be unable to acclimatise important components of the photosynthetic apparatus to high light. Nevertheless, these plants did not show symptoms of photoinhibition. High-light (HL) grown *WHIRLY1* knockdown plants showed clear signs of exposure to excessive irradiance such as a low epoxidation state of the violaxanthin cycle pigments and an early light saturation of electron transport. These responses were underlined by a very large xanthophyll cycle pool size and by an increased number of plastoglobules. Whereas zeaxanthin increased with HL stress, *α*-tocopherol, which is another lipophilic antioxidant, showed no response to excessive light. Also the content of the hydrophilic antioxidant glutathione showed no increase in W1 plants as compared to the wild type, whereas the flavone lutonarin was induced in W1 plants. HPLC analysis of removed epidermal tissue indicated that the largest part of lutonarin was presumably located in the mesophyll. Since lutonarin is a better antioxidant than saponarin, the major flavone present in barley leaves, it is concluded that lutonarin accumulated as a response to oxidative stress. It is also concluded that zeaxanthin and lutonarin may have served as antioxidants in the *WHIRLY1* knockdown plants, contributing to their survival in HL despite their restricted HL acclimation.

**Supplementary Information:**

The online version contains supplementary material available at 10.1007/s00425-023-04227-8.

## Introduction

Due to their sessile life style, plants are directly exposed to and in equilibrium with a large variety of environmental factors. Among them, sunlight is especially important, but this factor is also extremely variable, both in the short as well as in the long term. Plants are dependent on sunlight for photosynthesis and too little light will cause them to suffer. On the other hand, if the rate of light absorption in the photosynthetic apparatus exceeds the rate of consumption of light energy in the form of reducing equivalents in the photosynthetic dark reactions and/or poising mechanisms, an increased amount of reactive oxygen species (ROS) may be generated (Asada 2006; Fitzpatrick et al. 2022). Among the consequences of ROS formation is a damage to the photosynthetic reaction centers, especially photosystem II (PS II), resulting in a reduction of the photochemical quantum yield of PS II, which can be quantified using the chlorophyll fluorescence parameter *F*_*V*_/*F*_*M*_ (Demmig and Björkman 1987; Maxwell and Johnson 2000). Damage will especially occur when shade-acclimated plants are suddenly exposed to strong sunlight (Powles 1984; Anderson and Osmond 1987). However, most plant species will acclimate to high light by enhancing the capacity of the dark reactions (Anderson et al. 1995) and/or by employing photoprotective mechanisms such as non-radiative dissipation of excessive light, increased levels of antioxidants or by adjusting the redox state (Müller et al. 2001; Jahns and Holzwarth 2012; Asada 2006; Selinski and Scheibe 2019).

Dissipation of excess light energy as heat is one important short-term mechanism (from seconds to minutes) known as non-photochemical quenching (NPQ) of chlorophyll fluorescence, which occurs in the antenna system of PS II (Demmig-Adams and Adams 1996; Holzwarth et al. 2013). Energy partitioning to non-radiative dissipation can be easily quantified in intact leaves by determining the quantum yield of non-photochemical quenching (Φ(NPQ)) of chlorophyll fluorescence (Kornyeyev and Hendrickson 2007; Klughammer and Schreiber 2008). NPQ has been shown to depend on the formation of zeaxanthin from violaxanthin within the xanthophyll cycle (Bilger and Björkman 1990; Niyogi et al. 1998). In addition, zeaxanthin protects the thylakoid membrane against ROS-induced lipid peroxidation (Niyogi et al. 2001; Müller et al. 2001; Havaux et al 2007; Jahns and Holzwarth 2012). A further lipophilic antioxidant protecting against ^1^O_2_ is *α*-tocopherol (Spicher et al. 2017).

While these lipophilic antioxidants are predominantly important to detoxify ^1^O_2_, H_2_O_2_ generated at PS I is scavenged by hydrophilic antioxidants such as ascorbate and glutathione (Foyer and Noctor 2011; Hebbelmann et al. 2012). The latter compound is a small intracellular redox-active antioxidant molecule existing in two main stable forms: the reduced thiol (GSH) or the oxidized disulfide (GSSG) (Tausz et al. 2004). In plants growing under optimum conditions, the GSH/GSSG ratio is reported to have high values (Rahantaniaina et al. 2013; Bloem et al. 2015), with about 97% of the pool in the reduced form (Vanacker et al. 2000). Accordingly, a low GSH/GSSG ratio is often considered as a potential indicator for oxidative stress in plants, but may also be affected by other factors such as plant age (Tausz et al. 2004; Rahantaniaina et al. 2013; Bloem et al. 2015). Besides its direct role in ROS scavenging, glutathione is part of the *α*-tocopherol-ascorbate–glutathione triad (Szarka et al. 2012), maintaining the reduced state of tocopherol and thereby indirectly protecting cell membranes from oxidative damage (Hasanuzzaman et al. 2017). The synergistic antioxidant effect of the *α*-tocopherol-ascorbate–glutathione triad was also supported by the observation that the levels of these three antioxidants increased several fold in a coordinative manner under high-light conditions (Kanwischer et al. 2005).

In addition to ascorbate and glutathione, phenolic compounds such as flavonoids might also act as direct antioxidants (Havaux et al. 2007; Hernández et al. 2009; Agati et al. 2012; Nezval et al. 2017). In response to high-light stress, flavonoids bearing a catechol group, i.e. an ortho-dihydroxy group, at the flavonoid B-ring have been shown to accumulate in the vacuoles of mesophyll cells (Agati et al. 2009; Fini et al. 2011). These dihydroxy B-ring flavonoids, such as quercetin or luteolin derivatives, have a higher antioxidative activity than monohydroxy flavonoids such as kaempferol and apigenin derivatives (Rice-Evans et al. 1996; Agati et al. 2012; Alseekh et al. 2020).

When growing in high light, most plants, with the exception of obligate shade species, acclimate their photosynthetic capacities to prevent photoinhibition by reducing the excess excitation energy (Powles 1984; Gray et al. 1996). A recent study using barley plants with an RNAi-mediated knockdown of the multifunctional DNA-binding protein WHIRLY1 having a dual localization in chloroplasts and nucleus (reviewed by Krupinska et al. 2022; Taylor et al. 2023) showed that they cannot acclimate to high light. Acclimation is impaired both at the level of leaf morphology and at the level of the photosynthetic apparatus (Saeid Nia et al. 2022) suggesting that WHIRLY1 acts as a coordinator of acclimation processes at different levels of complexity.

Plants deficient in WHIRLY1 (W1) are expected to be prone to photoinhibition. Indeed, in the seedling stage these plants are bleached and accumulate ROS during photosynthesis as measured via electron paramagnetic spin resonance (EPR) spectroscopy on illuminated thylakoids (Swida-Barteczka et al. 2018). However, W1 plants did not show any decline in their maximum quantum yield of PS II after final chloroplast development during growth under high light (Kucharewicz et al. 2017). Hence, these plants are an excellent model system to study photoprotective mechanisms enabling plants to survive and avoid photodamage in the absence of acclimation of photosynthetic capacity. Therefore, in this study the WHIRLY1-deficient plants were used as a model to investigate the response of several antioxidative mechanisms in high-light exposed leaves.

## Material and methods

### Plant material and growth conditions

Grains of WHIRLY1-deficient plants prepared by RNAi-mediated knockdown of *HvWHIRLY1* (W1) (Krupinska et al. 2014) together with *Hordeum vulgare* L., cv. “Golden Promise” as wildtype (WT), were sown on soil (Einheitserde ED73, Einheitswerk Werner Tantau, Uetersen, Germany). Growth conditions were as described by Saeid Nia et al. (2022). Photosynthetic photon flux densities (PFD) incident on the leaf plane were 40–70 µmol m^−2^ s^−1^ for low light (LL) and 350–500 µmol m^−2^ s^−1^ for high light (HL), which corresponded to horizontal PFDs of 150–1000 µmol m^−2^ s^−1^, respectively. The incident irradiance on the adaxial and abaxial sides of the leaves was measured for every sampled individual primary leaf using a quantum sensor (Li-185 A, Li-Cor Biosciences, Lincoln, NE, USA). The area between 1.5 and 3 cm below the tip of primary foliage leaves, containing mature chloroplasts, was used for all measurements. Primary foliage leaves were used at different developmental stages, i.e. at 10, 15, and 19 days after sowing (das).

### Chlorophyll fluorescence measurements

Chlorophyll fluorescence was measured simultaneously with photosynthetic gas exchange (data shown in Saeid Nia et al. 2022) using a portable gas exchange fluorescence system GFS-3000 (Heinz Walz GmbH, Effeltrich, Germany). The instrument was set up with a 750 µmol min^−1^ air flow rate, a cuvette temperature of 21 °C, and 60% relative humidity. Attached primary leaves of both genotypes grown under HL and LL were measured at different das, reflecting different developmental stages. For the determination of F_V_/F_M_, plants were pre-darkened for at least 25 min.

The energy absorbed by PS II is partitioned into three main pathways which are expressed by their quantum yields, i.e. the quantum yield of photochemical energy conversion or Φ(II), the quantum yield of non-photochemical quenching or Φ(NPQ), and the sum of quantum yields of fluorescence and non-regulated heat dissipation of energy or Φ(NO) (Genty et al. 1996; Hendrickson et al. 2004; Klughammer and Schreiber 2008). These quantum yields were calculated as follows (Klughammer and Schreiber 2008).

$$\begin{gathered} \Phi \left( {{\text{II}}} \right) \, = \, \left( {F_{M} ^ \prime - F} \right)/F_{M} ^ \prime \hfill \\ \Phi \left( {{\text{NPQ}}} \right) \, = \, F/F_{M} ^ \prime - \, F/F_{M} \hfill \\ \Phi \left( {{\text{NO}}} \right) \, = \, F/F_{M} \hfill \\ \end{gathered}$$

ETR was calculated assuming an absorptance of 0.84 for both, WT and W1 leaves. For practical reasons, the true absorptance could not be determined. Since W1 leaves had reduced chlorophyll contents, especially at 10 das, their absorptance was lower than that of WT leaves, causing an overestimation of ETR in W1 leaves in comparison to WT. Absorptance was determined with a different set of primary leaves grown at HL at 10 das using an Imaging-PAM fluorometer (M-type, Heinz Walz GmbH). The data revealed that W1 leaves with chlorophyll contents corresponding to those used for ETR measurements had absorptances of 92% (HL) and 96% (LL), respectively, of the WT leaves. Hence, the ETR of W1 leaves may have been overestimated by 5–10%.

### Transmission electron microscopy (TEM) for the analysis of plastoglobules (PGs)

Ultrastructural analysis of chloroplasts in 10-day-old primary leaves of WT and W1 plants grown under LL and HL was done as described before by Saeid Nia et al. (2022). Four leaves were analyzed per condition. Segments from the mid part of primary foliage leaves were dissected and fixed with 1% glutaraldehyde in 200 mM Hepes, pH 7.4. Samples were post-fixed with 1% OsO_4_ prepared in 1.5% aqueous potassium ferricyanide, contrasted en-bloc with 2% aqueous uranyl acetate, then dehydrated with a graded ethanol series, followed by 100% acetone, next progressively infiltrated with epon resin and then heat polymerised. Ultrathin 80-nm sections were contrasted with saturated aqueous uranyl acetate and lead citrate, and inspected in a Tecnai G2 Spirit BioTWIN transmission electron microscope (FEI, now Thermo Fisher Scientific) equipped with an Eagle 4k × 4k CCD camera and TIA software (both FEI).

### High-performance liquid chromatography (HPLC) analysis of pigments

From the area between 1.5 and 3 cm below the tip, 1 cm long leaf segments were cut from plants in the climate chamber under the growth irradiance. After measuring the segments’ widths, these samples were immediately frozen in liquid nitrogen and stored at − 80 °C till the time of extraction. Chlorophyll and carotenoid extraction and separation by HPLC have been described by Saeid Nia et al. (2022).

As described by Nichelmann et al. (2016), for calibrating the detector, pure extracts of carotenoids (except antheraxanthin) were prepared through thin-layer chromatography (modified after Lichtenthaler and Pfister 1978), and their concentrations were determined by spectrophotometry using the extinction coefficients provided by Davies (1976). The concentrations in the standard solutions were expressed in pmol mL^−1^ and recalculated as nmol cm^−2^ for the leaf samples. The epoxidation state of the xanthophyll-cycle pigments (EPS) was calculated according to Thayer and Björkman (1990) as (*V* + 0.5 *A*)/(*V* + *A* + *Z*), with *V*, *A* and *Z* denoting the contents of violaxanthin, antheraxanthin and zeaxanthin, respectively.

### HPLC analysis of tocopherols

Leaf segments were prepared and stored at − 80 °C as explained above. To extract tocopherols, frozen segments of a total of *n* = 6 leaves for each genotype grown under LL and HL at 10, 15 and 19 das, and from three independent experiments each comprising 2 leaves were ground with 400 µL HPLC-grade n-heptane together with 5–6 glass beads in a Geno/Grinder (Type 2000; SPEX CertiPrep, Munich, Germany). Afterwards, samples were briefly centrifuged and kept at − 20 °C overnight. The next day, samples were mixed and supernatants were collected after centrifugation for 10 min at 16,000 *g* at 4 °C (Kendro Biofuge Fresco, Osterode, Germany). After a second mixing the centrifugation was repeated once more. Finally, 20 µL of supernatants were used for chromatographic analysis of tocopherols using a Shimadzu HPLC system equipped with an RF-10A XL fluorescence detector, 10-series (Shimadzu Corporation, Kyoto, Japan). Tocopherol separation was done using a Lichrospher Si 60 column (5 µm/250–4 mm, Merck, Darmstadt, Germany) and an isocratic system as described before (Sickel et al. 2012). The pump (LC-10AT VP) delivered a constant flow of 1 mL min^−1^ of the eluent (n-heptane and isopropanol (99/1, *v*/*v*)). Tocopherols were quantified using an external standard solution of a mixture of tocopherols and tocotrienols of known concentrations (Merck KG, Darmstadt, Germany).

### Analysis of leaf flavonoid content and composition by HPLC

Leaf segments from 1.5 cm below the tip of primary leaves of WT and W1 plants grown under LL or HL were sampled and frozen in liquid nitrogen as described above. For each genotype grown under LL and HL in total 20–23 leaves from three independent experiments, each comprising 6–8 leaves, were used. Samples were kept in the freezer at − 80 °C until HPLC analysis. To prepare samples for HPLC, 250 µL of the cold (4 °C) extraction buffer consisting of 49.5% (*v*/*v*) distilled water and 49.5% (*v*/*v*) methanol with an addition of 1% (*v*/*v*) concentrated HCl (Merck) was added to each sample. The samples were homogenized for 3 min at 1700 strokes min^−1^ in a Geno/Grinder 2000 (SPEX CertiPrep) followed by 5 min centrifugation at 16,000 × *g* at 4 °C (Biofuge Fresco). Resulting pellets were resuspended twice in 250 µl of extraction buffer and centrifuged again. All supernatants were collected and centrifuged for 10 min with 10,000 × *g* at 4 °C. 500 µL of each supernatant was purified through 0.45 µm filters (National Scientific, Rockwood, USA).

30 µL of the extracted solution were injected in an HPLC system with a diode array detector (SCL-10AT VP, SIL-10AD 145 VP, LC-10AT VP, FRC-10A, SPD-M10A VP, Shimadzu) and separated on a LiChrospher 100 RP-18 column (4 ∗ 250 mm, 5 µm particle size, Merck, Germany). Eluent A, 0.01% phosphoric acid, and eluent B, 90% methanol with 0.1% phosphoric acid, were used in this system as mobile phases at a flow rate of 1 mL min^−1^. The gradient started with 80% eluent A for the first 12 min followed by a linear decrease in the proportion of eluent A to 55% for 28 min. Afterwards, eluent B was increased linearly to 100% and stayed constant for 12 min. Flavonoids and hydroxycinnamic acids (HCA) were detected at 313 nm and the chromatograms were analysed using LC Solution software (Shimadzu). Representative chromatograms are presented in Fig. S1. Flavonoid contents were expressed in AU s per leaf area without further quantification.

### Identification of flavonoids by mass spectrometry

For this analysis, 28–31 leaves from each genotype grown under LL and HL and from four independent experiments, each comprising 7–8 leaves were used. To identify the flavonoids of interest soluble semi-polar metabolites were extracted from liquid nitrogen-frozen leaf segments in 2-mL screw cap reaction tubes (Sarstedt, Germany). After weighing the samples, ZrO_2_ 58% beads (RIMAX ZS-R Ø 1.0–1.2 mm, Mühlmeier, Germany) and 400 µL of LC–MS grade methanol per 100 mg of fresh tissue were added. Sample thawing prior to methanol addition was avoided. Sample grinding and extraction were done in a Precellys homogenizer (Bertin Instruments, France) at 8000 s^−1^, with two cycles of 10 s each. Following centrifugation (22,500×*g*, 4 °C, 10 min), the supernatant was recovered into a clean tube and the pellet was resuspended in the same volume of methanol per 100 mg of fresh weight. After mixing, the second supernatant was recovered by centrifugation; the two supernatants were combined and stored at − 20 °C. Before analysis, 80 μL-aliquots from the methanolic extracts were mixed with 20 μL of 0.5% (*v*/*v*) formic acid, incubated overnight (− 20 °C), and centrifuged (22,500×*g*, 4 °C, 10 min) to remove precipitates.

For the identification of flavonoids, the extracts were analyzed via Reversed Phase Ultra Performance Liquid Chromatography-Photodiode Array-Electrospray Ionization-Ultra-High-Resolution-Quadrupole Time Of Flight-tandem Mass Spectrometry (RP-UPLC-PDA-ESI-UHR-QTOF-MS/MS) as described in Garibay-Hernández et al. (2021). The analysis was carried out using an Acquity UPLC system (Waters, Germany), equipped with an Acquity PDA *eλ* detector, coupled to a maXis Impact ESI-QTOF MS (Bruker Daltonik GmbH, Germany). The Compass HyStar 3.2 SR2 software (Bruker Daltonik GmbH) was used to operate and coordinate LC-PDA-MS data acquisition. Data processing, analysis, and compound identification were performed using the software packages Compass Data Analysis V4.4 and Metaboscape 5.0 (Bruker Daltonik GmbH, Germany). Compound identity was confirmed by exact mass (error < 5 ppm), isotopic pattern, MS/MS fragmentation, and PDA spectra (Table 1). Commercial standards were employed when available.

**Table 1:** Flavonoid identification by mass spectrometry

Annotation	*λ* max* (nm)*	Molecular formula	Monoisotopic mass	Precursor ion	Calculated *(m/z)*	Measured *(m/z)*	Fragment ions	Measured *(m/z)*
Lutonarin^a^	268 (s), 348	C_27_H_30_O_16_	610.1534	[M + H]^+^	611.1606	611.1636	[Lut + H + Hex]^+^	449.1093 (29.2)
							[Lut + H + C_2_H_2_O]^+^	329.0657 (98)
							[Lut + H + C]^+^	299.0551 (100)
							[Lut + H]^+^	287.0547 (4.1)
Saponarin^b^	269, 336	C_27_H_30_O_15_	594.1585	[M + H]^+^	595.1657	595.1681	[Api + H + Hex]^+^	433.1139 (58.6)
							[Api + H + C_2_H_2_O]^+^	313.0711 (100)
							[Api + H + C]^+^	283.0607 (82.6)
							[Api + H]^+^	271.059 (4.4)

The flavonoids of interest were identified by RP-UPLC-PDA-ESI-HR-QTOF-MS/MS

^a^Isoorientin 7-O-glucoside; Luteolin 6-C-glucosyl-7-O-glucoside

^b^Isovitexin 7-O-glucoside; Apigenin 6-C-glucosyl-7-O-glucoside

Compound annotations were confirmed by exact mass (mass accuracy below 5 ppm), isotopic pattern, MS/MS fragmentation, and PDA spectra. The percentage of relative intensities of the MS fragment ions are indicated in parenthesis. The PDA- and MS-spectra were compared to the available literature (Brauch et al. 2018; Garibay-Hernández et al. 2021; Piasecka et al. 2015). The identification of saponarin was also confirmed with a commercial standard (1238S, Extrasynthèse, France)

Abbreviations: *Api* Apigenin, *Hex* hexosyl moiety (neutral loss 162.05), *Lut* Luteolin, *m/z* mass to charge ratio, *s* shoulder

### Localization of leaf flavonoids

To localize the main flavonoids of the leaves, the leaf abaxial epidermis was separated from the mesophyll by a careful vertical cut in the adaxial epidermis and by gently pulling the epidermis and the remainder of the leaf (leaf plus adaxial epidermis) from each other. For localization of the leaf flavonoids, three samples from three independent experiments for each genotype grown under LL and HL were used. For each sample, three to five segments of a size of 1–2 cm^2^ of the epidermis and of the remaining part (leaf lacking the abaxial epidermis), respectively, were pooled, frozen in liquid nitrogen, and kept in a freezer at − 80 °C until HPLC analysis. The flavonoid content of samples was calculated per leaf segment area.

To estimate the flavonoid content in the mesophyll, the flavonoid content of the adaxial epidermis was calculated. To this end, it was assumed that the content of flavonoids in the two epidermal tissues would be proportional to their epidermal absorbance. The latter was determined by chlorophyll fluorescence analysis from both sides of the leaves using a combination of a UVA-PAM fluorometer (Gademann Instruments, Würzburg, Germany; Bilger et al. 2001) and a Mini-PAM fluorometer (Heinz Walz GmbH). After normalization of the fluorescence signals to the signal obtained with a blue plastic film (Walz), the fluorescence signal determined with the UV-A measuring beam (F(UV-A)) was divided by that of the red beam (F(R)). UV-A transmittance and absorbance were calculated using the F(UV-A) to F(R) ratios of epidermis-free leaves of *Vicia faba* as a reference for a signal obtained with 100% epidermal transmittance. The flavonoid content of the adaxial epidermis was calculated from the HPLC results for the abaxial epidermis using the slope of the linear regression between the absorbances of both leaf sides (Fig. S2). The result was subtracted from the flavonoid content of the remaining segments to calculate the flavonoid content of the mesophyll.

### Gene expression analysis by quantitative RT-PCR

RNA was isolated from a pool of eight leaf segments excised from the middle part of primary leaves using the peqGOLD-TriFast reagent (Peqlab Biotechnology, Erlangen, Germany) as described (Krupinska et al. 2019). Thereafter, RNA concentration was quantified by a Nanodrop instrument 200 (Thermo Scientific). 500 ng of extracted RNA was used to synthesize cDNA using reverse a transcriptase kit (Quanti Tect^®^, Qiagen, Hilden, Germany) according to the protocol provided by the manufacturer. By using gene-specific primers (details about the gene accession numbers and designed primers can be found in the Supplementary Data, Table S1), gene expression was analysed by quantitative PCR as described by Krupinska et al. (2019) and normalized to the mRNA level of the ADP-ribosylation factor 1 as the reference gene (Rapacz et al. 2012). The expression levels of genes of interest were analysed from three independent experiments (each comprising 8 leaves) and each with three technical replicates per sample using the Rotor-Gene Q Series Software (version 2.0.2.4, Qiagen).

Quantification of transcript levels was performed relative to the expression level in LL-grown wild-type plants (as the control) by the “delta-delta *C*_*T*_ method” as described by Livak and Schmittgen (2001).

### Determination of the glutathione content and its redox state

For each genotype grown under either LL or HL, a pool of nine primary leaves in total from three independent experiments each comprising three leaves, respectively, were ground with 5–6 glass beads in a Geno/Grinder (Type 2000; SPEX CertiPrep) to a fine powder. Liquid nitrogen was continuously used to avoid the thawing of frozen leaves or their powder. Thereafter, about 50 mg of the powder of each sample was collected in new tubes in the presence of liquid nitrogen.

Total glutathione and GSSG concentrations were determined using a glutathione colorimetric detection kit according to the manufacturer’s protocol (Invitrogen™ Glutathione Colorimetric Detection Kit, Thermo Fisher Scientific). The absorbance of samples (each with three technical replications) together with a dilution series (to perform a standard curve) of standards (provided in the kit) were measured at 405 nm by a plate reader (TECAN-infinite M200 PRO, TECAN Austria GmbH) in a kinetic assay every minute for 10 min.

To analyze the data, the average of triplicate absorbance measurements for each experimental sample, standard, and background at each time point was calculated and plotted against the incubation time. The slope from the linear part of each curve was determined. The background slope was subtracted from the slope of all standards and samples. Thereafter, the slopes of the standards were plotted against their concentration, and then the slope from the linear part of this curve was used to determine the concentration of total glutathione and GSSG, respectively, in the experimental samples. The amounts of total glutathione and GSSG were calculated per dry weight of the sample pellets. To calculate the reduced glutathione concentration, the concentration of oxidized glutathione was subtracted from the total glutathione concentration.

### Statistical analysis

Sigmaplot 13 (Systat Software GmbH, Erkrath, Germany) or GraphPad PRISM (Prism 9 for Windows, version 9.2.0 (332), GraphPad Software, San Diego, California USA) was used for statistical analysis. Two-way ANOVA (with the factors genotype and growth irradiance or age) or three-way ANOVA (with the factors genotype, growth irradiance, and age) were used to compare among groups, and in case of significant differences, the Holm-Sidak method was used for comparison of the means. In this study, all the experiments were repeated at least two times, except the HPLC analysis for the pigments of the photosynthetic apparatus.

## Results

### Photosynthetic electron transport rate (ETR)

**Fig. 1:**
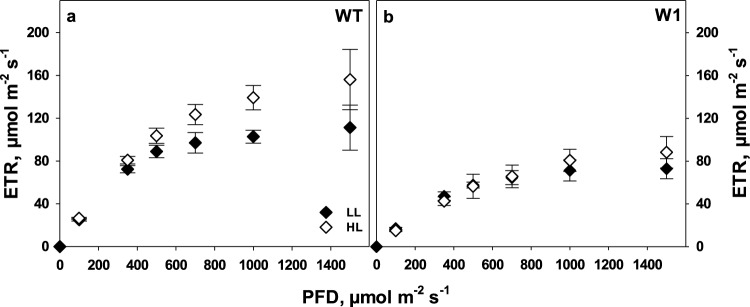
Electron transport rates (ETR) in WT (**a**) and W1 (**b**) plants grown under low (LL, filled symbols) and high light (HL, open symbols) as a function of incident irradiance (PFD). Measurements were done in the presence of 1500 ppm CO_2_ at 10 days after sowing (das). Depicted values are means ± standard deviation (SD) of *n* = 9–11 leaves in total from three independent experiments each comprising 3–4 leaves

Previous gas exchange measurements had revealed that HL-grown WHIRLY1-deficient transgenic plants (W1) were unable to acclimate their photosynthetic capacity to a higher irradiance in contrast to wild-type (WT) plants (Saeid Nia et al. 2022). When electron transport rates (ETR) as determined by chlorophyll fluorescence measurements were compared between WT and W1 plants, a similar difference was observed (Fig. 1). Whereas the maximal electron transport rates of W1 plants grown under HL or LL did not differ (*P* = 0.093), leaves of WT plants grown under HL showed significantly higher ETR than LL-grown WT plants (Fig. 1). Comparing WT to W1 plants, WT plants showed significantly higher maximum ETR in both LL and HL (Fig. 1).

### The maximal quantum yield of photosystem II

**Fig. 2:**
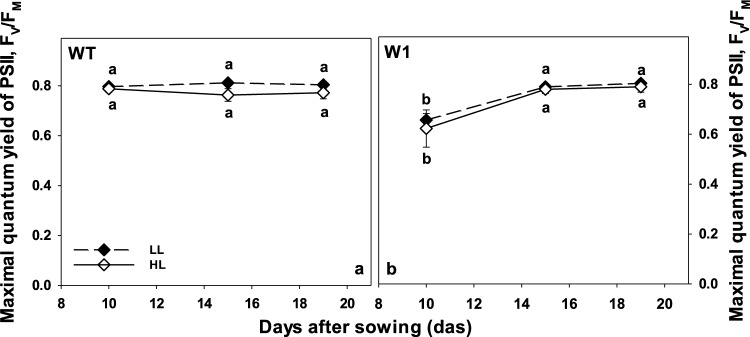
Maximal quantum yield of PS II, *F*_*V*_/*F*_*M*,_ measured in WT (**a**) and W1 (**b**) plants grown under low light (LL, filled symbols) and high light (HL, open symbols) as a function of plant age. Depicted values are means ± standard deviation of a total of *n* = 9–11 leaves in total from three independent experiments, each comprising 3–4 leaves. The letters indicate statistically different values at a significance level of *P* = 0.05, as determined by two-way ANOVA with time and genotype as factors, followed by pairwise multiple means comparisons with the Holm-Sidak method

The maximal quantum yield of PS II (*F*_*V*_/*F*_*M*_) in leaves of LL-grown WT plants stayed stable from 10 to 19 das (Fig. 2, *P* > 0.9999). The small decrease in *F*_*V*_/*F*_*M*_ in HL-grown WT from 10 to 15 and 19 das was not significant (Fig. 2a).

Similar to previous results (Kucharewicz et al. 2017), in leaves of 10-day-old W1 plants, *F*_*V*_/*F*_*M*_ in both LL- and HL-grown plants was significantly lower compared to the values of the WT and also compared to those of W1 plants at 15 and 19 das.

Progressing development of W1 plants from day 10 to day 15 was accompanied by a significant increase in the maximal quantum yield of PS II (Fig. 2b) reaching a value commonly observed in unstressed plants (Björkman and Demmig 1987) despite their inability to acclimate their photosynthetic capacity to high growth irradiance (Saeid Nia et al. 2022). This suggests that their lower photosynthetic capacity was compensated through other mechanisms. To investigate this, the fate of excitation energy in PS II of HL-grown WT and W1 plants, respectively, was analyzed in more detail at 10 and 15 das.

### The fate of excitation energy in PS II

**Fig. 3:**
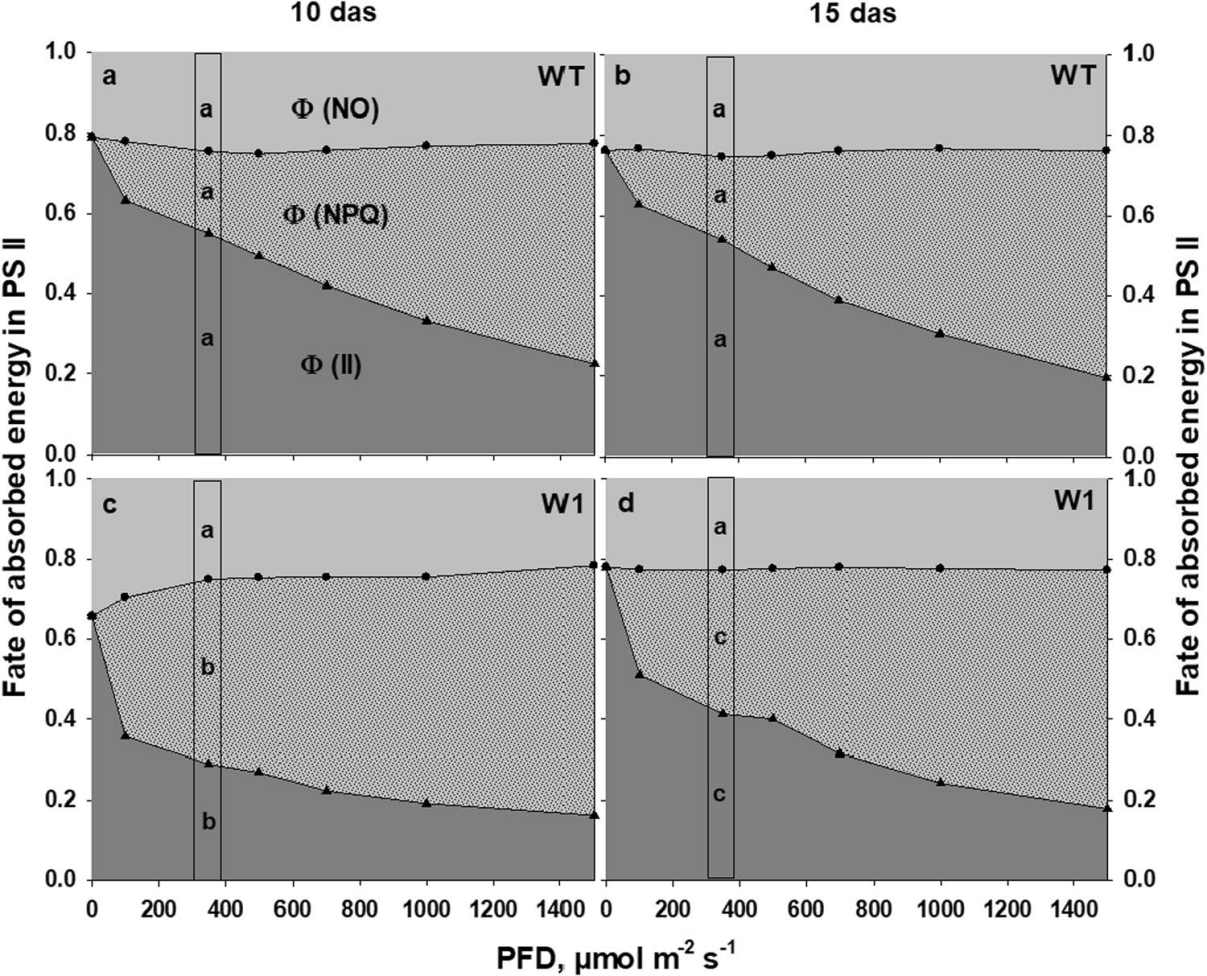
The fate of excitation energy in PS II as a function of incident PFD in HL-grown WT (**a**, **b**) and W1 plants (**c**, **d**) at 10 and 15 das. Measurements were done in the presence of 1500 ppm CO_2_. Depicted values are means of a total of *n* = 9–11 leaves in total from three independent experiments each comprising 3–4 leaves. The boxes highlight the fate of absorbed energy in PS II at the incident light of 350 µmol m^−2^ s^−1^, which is similar to the growth irradiance of HL-grown plants. The letters indicate statistically different values between WT and W1 leaves at 10 and 15 das for each of the different quantum yields separately at a significance level of *P* = 0.05, as determined by two-way ANOVA with das and genotype as factors, followed by pairwise multiple means comparisons with the Holm-Sidak method

The analysis of the fate of absorbed energy in PS II revealed that 10-day-old W1 plants had a lower quantum yield of photosynthesis (Φ(II)) (Fig. 3c) in comparison to WT plants (Fig. 3a). However, their lower Φ(II) was accompanied by a higher quantum yield of non-photochemical quenching (Φ(NPQ)).

No specific changes were detected in Φ(II) and Φ(NPQ) of WT plants between 10 and 15 das (Fig. 3b). In contrast, Φ(II) increased and Φ(NPQ) decreased in W1 plants from 10 to 15 das (Fig. 3d).

Since the HL-grown plants received an irradiance of about 350 µmol m^−2^ s^−1^ during growth, the fate of absorbed energy at this irradiance was analyzed further in WT and W1 plants at 10 and 15 das (boxes in Fig. 3). The two-way ANOVA analysis showed that there were no significant differences in the quantum yield of PSII or Φ(NPQ) between WT plants at 10 and 15 das.

While Φ(II) was significantly lower in WHIRLY1-deficient plants than in WT plants at 10 and 15 das, the Φ(NPQ) was significantly higher. Moreover, as the development of W1 plants progressed between 10 and 15 das, a significant increase in Φ(II) was accompanied by a significant decrease in Φ(NPQ). Intriguingly, there were no significant differences between non-regulated energy dissipation (Φ(NO)) of WT and W1 plants at 10 and 15 das.

### Ultrastructural analyses revealed an accumulation of plastoglobules in high-light-grown W1 leaves

**Fig. 4:**
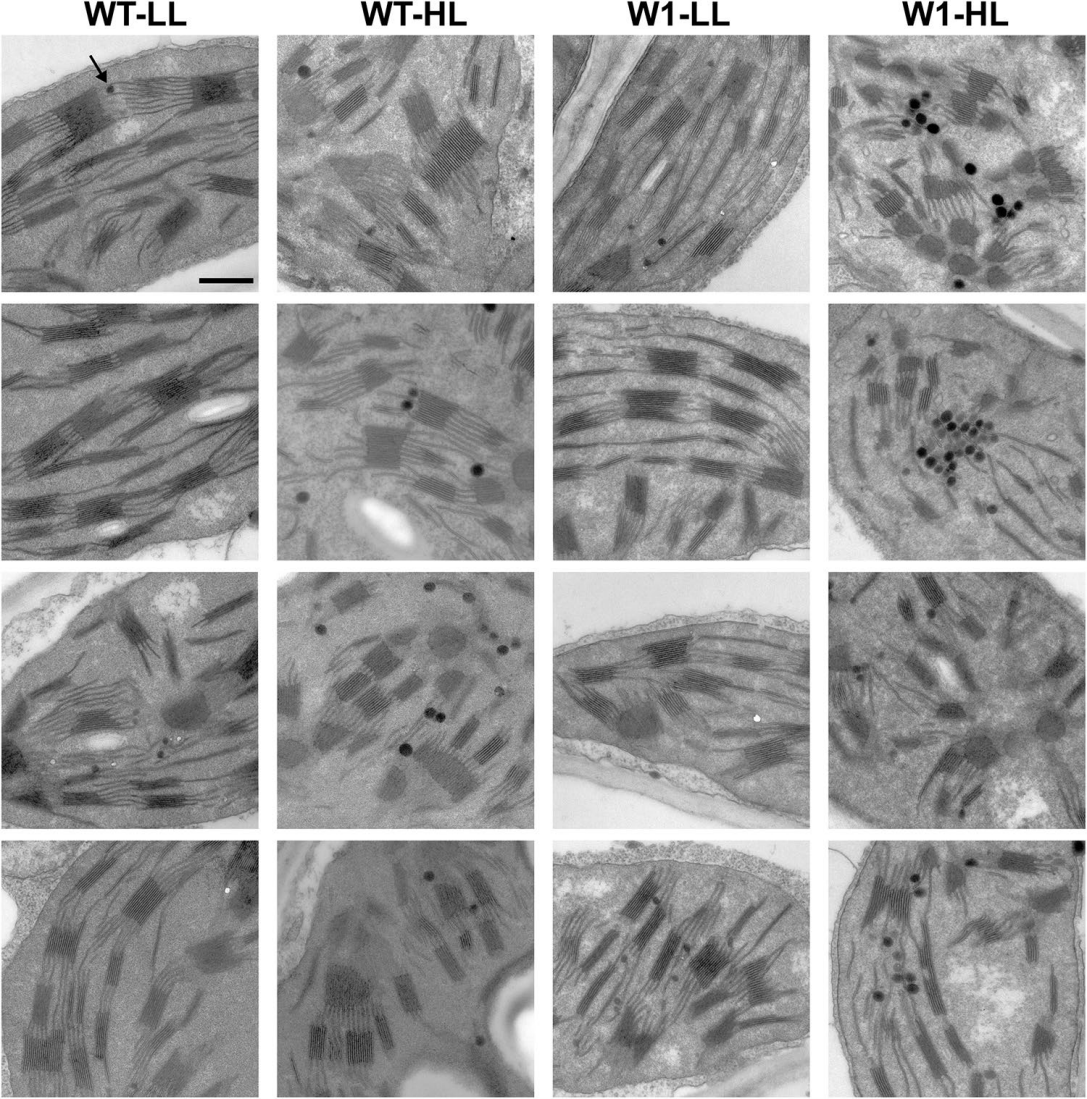
Ultrastructural analysis of chloroplasts in primary foliage leaves of WT and W1 plants grown under high light (HL) or low light (LL) for 10 days. Plastoglobules (arrow) appear as electron-dense granules associated with stroma thylakoid membranes. Under HL, plastoglobules increased in size and abundance. Four images per condition (selected among images of two leaves, out of four leaves analysed) are shown to illustrate the diversity of plastoglobule phenotype observed on thin sections. Scale bar, 500 nm

Oxidative stress in chloroplasts is accompanied by an accumulation of plastoglobules (Austin et al. 2006). To compare the abundance and size of these lipid particles, the chloroplast ultrastructure of WT and W1 plants grown for 10 days at either HL or LL was analyzed (Fig. 4). In WT and W1 plants grown under LL, the abundance of plastoglobules, their size (maximum diameter ~ 80 nm) and their localization along stroma thylakoids were similar. In HL-grown plants, plastoglobules were larger (maximum diameter ~ 130 nm) and in W1 plants also notably more abundant. A particularly prominent feature of increased abundance were row-like clusters of plastoglobules that were lined up along adjacent stroma thylakoid membranes. In accordance with the data obtained by characterization of photosynthesis (Figs. 1, 2, 3), these observations suggest that W1 plants exposed to excessive light suffered from oxidative stress (Bréhélin et al. 2007; Rottet et al. 2015).

### Pigments of the photosynthetic apparatus

In view of the well-known formation of zeaxanthin in response to excessive excitation energy and its role in the mediation of NPQ (Jahns and Holzwarth 2012), the carotenoid content of WT and W1 leaves at 10 and 15 das was analyzed. For comparison, the chlorophyll content of plants was also analyzed since it represents the progress of the development of W1 plants from day 10 to day 15.

**Fig. 5:**
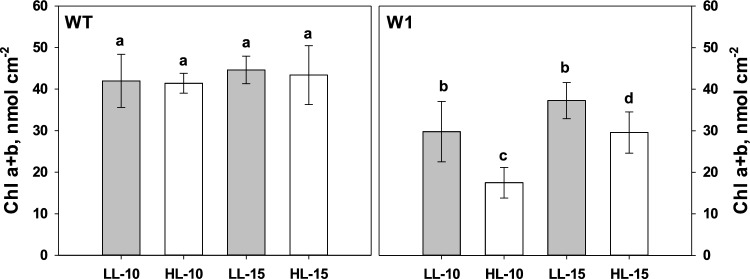
Chlorophyll content on 10 and 15 das of WT (**a**) and W1 (**b**) plants grown at low light (LL) and high light (HL), respectively. Columns are means ± standard deviation from *n* = 6 samples from one experiment. The letters indicate statistically different values at a significance level of *P* = 0.05, as determined by three-way ANOVA, followed by pairwise multiple means comparisons by the Holm-Sidak method

As shown previously by Kucharewicz et al. (2017) and Saeid Nia et al. (2022), the chlorophyll content of WT plants, did not change from day 10 to day 15 (Fig. 5a), whereas it increased in the case of W1 plants significantly at HL (Fig. 5b). This indicates that the development of chloroplasts in W1 plants is delayed, in accordance with previous observations (Krupinska et al. 2019).

**Fig. 6:**
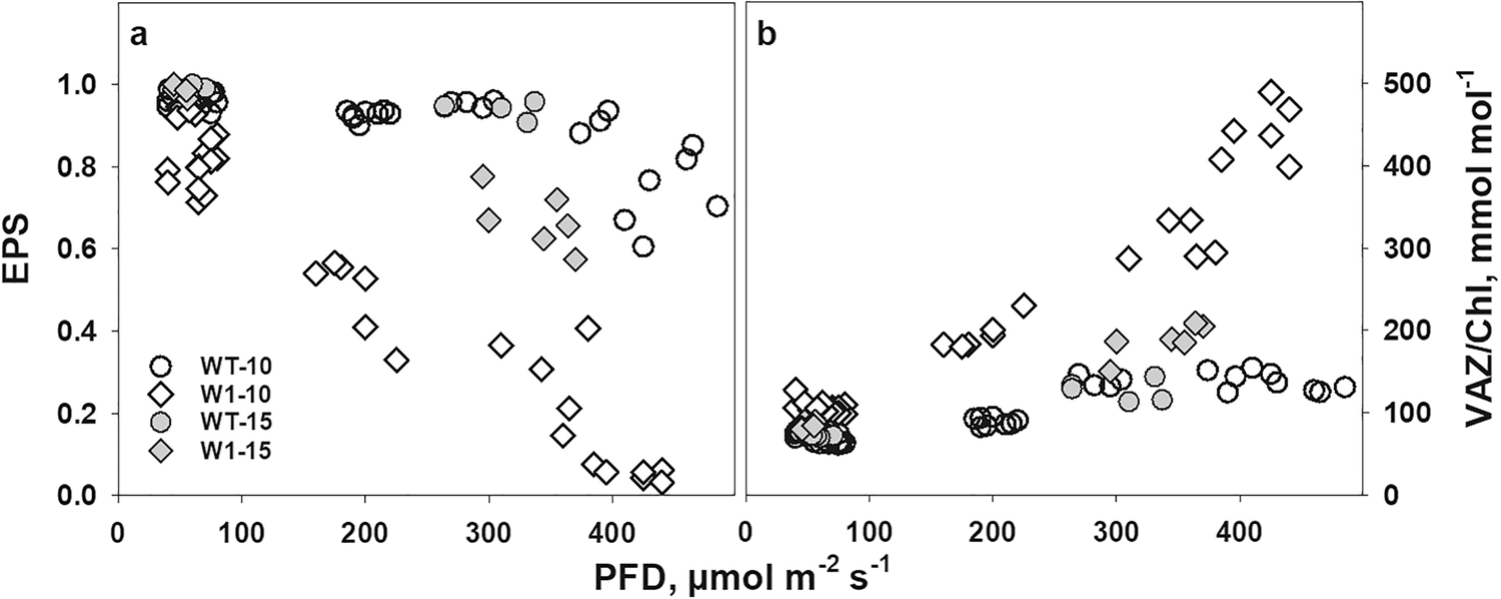
The epoxidation state (EPS) (**a**) and the pool size of the violaxanthin cycle (VAZ) per chlorophyll (**b**) in single WT and W1 leaves as a function of the growth irradiance (PFD) incident on these leaves. Circles and diamonds denote WT and W1 plants, respectively. Open and filled symbols denote 10- and 15-day-old plants, respectively. Data are taken from two experiments, one in which sampling was only done at 10 das, and another one in which sampling was at 10 and 15 das

Under excessive light, zeaxanthin is formed in chloroplasts causing a low epoxidation state (EPS) of the xanthophyll cycle pigments which is an in vivo indicator of excessive PFD (Demmig-Adams et al. 1990; Ort 2001). In line with the photosynthesis measurements taken ex situ (Saeid Nia et al. 2022, Fig. 1), EPS determined in situ under growth conditions significantly declined in W1 plants to values close to zero with increasing growth irradiance (Fig. 6a). In both WT and W1 plants, the xanthophyll cycle pool (*V* + *A* + *Z*) size increased significantly with increasing light. However, in leaves of W1 plants, the VAZ pool was considerably larger than in the WT (*p* < 0.0001) (Fig. 6b). Also lutein increased strongly with increasing PFD in the W1 plants (Fig. S3). WHIRLY1-deficient plants had a significantly lower epoxidation state of the cycle and larger VAZ pool size in comparison to WT plants even under LL conditions (Fig. 6a and b).

As chloroplast development in the W1 plants progressed, indicated by the higher chlorophyll content at 15 das in comparison to 10 das (Fig. 5b), EPS increased (*p* < 0.0001) (Fig. 6a), and the xanthophyll cycle pigment pool size decreased (*p* < 0.0001) (Fig. 6b). Nevertheless, at 15 das, HL-grown W1 plants still showed a significantly larger VAZ pool size (Fig. 6b) and significantly lower EPS compared to the WT (Fig. 6a). These data are in accordance with the results obtained with barley WT and W1 plants grown in continuous light (Swida-Barteczka et al. 2018).

**Fig. 7:**
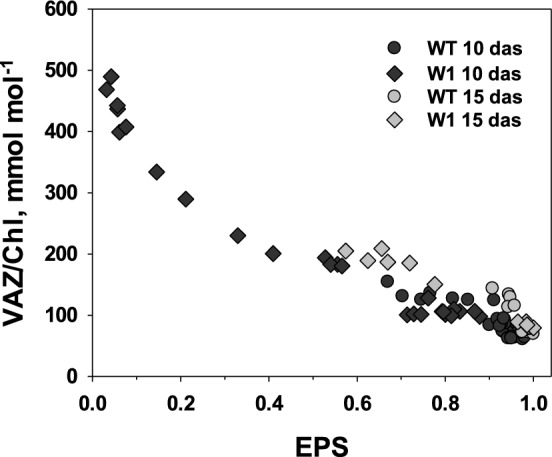
VAZ/Chl in single leaves of WT and W1 plants grown under different irradiance as a function of the violaxanthin cycle epoxidation state (EPS). Data are from the same experiments as those shown in Fig. 6

It has been proposed that the VAZ pool size increases as a function of excessive PFD (Bilger et al. 1995). To investigate this relationship, the VAZ pool size was plotted as a function of EPS. The data for both genotypes, WT and W1 collected at 10 das, followed a single common function (Fig. 7). With further development in the W1 plants, the decline in VAZ/Chl was coordinated with the increase in EPS, causing the LL data points from 15 das to fall on the same relationship as data from 10 das. Only samples from HL showed a slightly enhanced VAZ pool size at 15 das in both genotypes, WT and W1, in comparison to 10 das.

### Expression of genes required for zeaxanthin formation and its epoxidation

Relative expression levels of genes encoding *β*-carotene hydroxylase-1 (*HvbcHYD*) and zeaxanthin epoxidase (*HvZEP*) were measured.

**Fig. 8:**
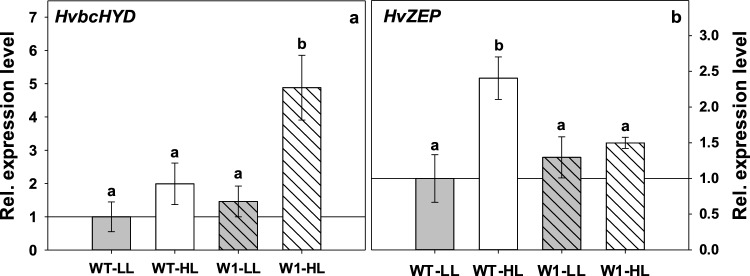
Relative expression level of *β*-carotene hydroxylase-1, *HvbcHYD* (**a**) and zeaxanthin epoxidase, *HvZEP* (**b**) in WT and W1 grown under low (LL, grey bars) and high light (HL, grey bars) at 10 das. Columns are means ± standard deviation from *n* = 3 samples from three independent experiments each comprising one sample. Each sample was a pool of 8 leaves. Quantification of transcript levels was performed relative to the expression level in LL-grown WT plants as the control (WT-LL is set at 1.0). The letters indicate statistically different values at a significance level of *P* = 0.05, as determined by two-way ANOVA, followed by pairwise multiple means comparisons by the Holm-Sidak method

HvbcHYD is the key enzyme of zeaxanthin biosynthesis (Sun et al. 1996). As expected, expression of *HvbcHYD* was increased in WT plants in HL with respect to LL, however, the increase was not strong enough to be significant. In comparison, the relative expression level of *HvbcHYD* in HL-grown W1 plants was significantly higher than in LL-grown W1 plants as well as in HL-grown WT plants (Fig. 8a).

HL-grown WT plants also showed significantly higher *HvZEP* expression in comparison to those grown in LL, while no significant difference in the expression of *HvZEP* was detectable between transgenic plants grown in LL or HL (Fig. 8b).

### Tocopherol content of leaves

**Fig. 9:**
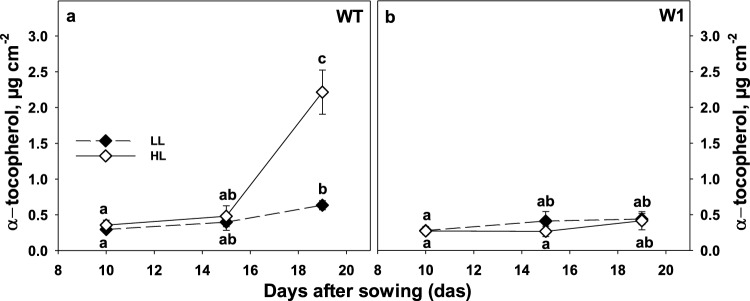
*α*-tocopherol content of leaves in WT (**a**) and W1 (**b**) plants grown under low light (LL, filled symbols) and high light (HL, open symbols) as a function of days after sowing (das). Depicted values are means ± standard deviation of *n* = 6 samples from in total three independent experiments each comprising two leaves. The letters indicate statistically different values at a significance level of *P* = 0.05, as determined by three-way ANOVA with genotype, irradiance, and das as factors, followed by pairwise multiple means comparisons by the Holm-Sidak method (see Table S2)

Tocopherols are important lipophilic antioxidants, which might protect the thylakoid membrane during growth in HL (Munné-Bosch and Alegre 2002). Unexpectedly, the *α*-tocopherol content did not differ between WT and W1 plants when grown for 10 or 15 das in either LL or HL (Fig. 9a and b, see also Table S2). In contrast, at a later stage of development, i.e. after 19 das, the *α*-tocopherol content increased in WT leaves, whereas it stayed constant in W1 leaves (Fig. 9).

In addition to the predominant *α*-tocopherol, also minor contents of *β*-tocopherol and γ-tocopherol as well as the total tocopherol content of WT and W1 plants grown under LL and HL at different developmental stages were analyzed (Table S2). The tendency of the data matched that obtained for *α*-tocopherol (see Table S2).

### HPLC analysis of flavonoid content

**Fig. 10:**
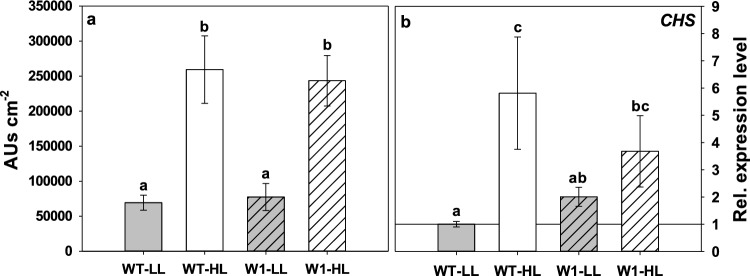
**a** The total amount of flavonoids in leaves of WT and W1 plants grown under low (LL, grey bars) and high light (HL, white bars) at 10 das expressed as the sum of all flavonoid peaks detected at 313 nm in the chromatograms. The data show means ± standard deviation of *n* = 20–23 leaves in total from three independent experiments each comprising 6–8 leaves. **b** The relative expression level of the gene encoding chalcone synthase (*CHS*) in WT and W1 plants grown under LL and HL at 10 das. Quantification of transcript levels was performed relative to the expression level in LL-grown WT plants as the control and therefore WT-LL is set at 1.0. Columns are means ± standard deviation from *n* = 3 samples from three independent experiments each comprising one sample. Each sample was a pool of 8 leaves. The letters indicate statistically different values at a significance level of *P* = 0.05, as determined by two-way ANOVA with genotype and irradiance as factors, followed by pairwise multiple means comparisons by the Holm-Sidak method

Among the hydrophilic antioxidants assumed to support photoprotection in plants are the flavonoids. Accordingly, HPLC analysis showed a significant increase in the total content of flavonoids (calculated as the sum of the area of the six highest flavonoid peaks (*P*_2_ to *P*_7_) corrected for the leaf area) as a response to HL in both genotypes. However, there was no significant difference between WT and W1 plants, neither under LL (analyzed with two-way ANOVA) nor under HL (Fig. 10a).

Nevertheless, the relative expression level of the gene encoding one of the key enzymes in flavonoid biosynthesis, chalcone synthase (*CHS*), was significantly enhanced in WT plants (*P* = 0.001) during growth in HL (Fig. 10b). The tendential increase of the expression of *CHS* in W1 plants in response to HL was not significant (*P* = 0.137).

### Flavonoid composition of leaves

**Fig. 11:**
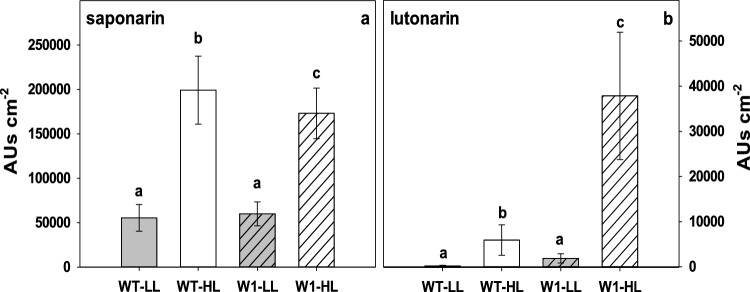
Leaf content of saponarin (**a**) and lutonarin (**b**) expressed as HPLC peak area in WT and W1 grown under low (LL, grey bars) and high light (HL, white bars) at 10 das. The data show the means ± standard deviation of *n* = 28–31 leaves in total from four independent experiments each comprising 7–8 leaves. The letters indicate statistically different values at a significance level of *P* = 0.05, as determined by two-way ANOVA with genotype and irradiance as factors, followed by pairwise multiple means comparisons by the Holm-Sidak method

Whereas the total flavonoid content did not differ between WT and W1 leaves, the flavonoid composition differed considerably between WT and W1 plants in HL conditions (Fig. 11). The two prominent peaks in HPLC chromatograms of the HL grown W1 plants (see representative chromatograms in Fig. S1) were identified based on their UV and MS spectra (Table 1). The highest peak in both genotypes under either HL or LL was confirmed as saponarin (apigenin-6-*C*-glucosyl-7-*O*-glucoside). In line with the total amount of flavonoids, HL-grown plants showed a significantly higher content of saponarin than LL-grown plants. Moreover, the amount of saponarin was 10% higher in HL-grown WT plants than in W1 plants (Fig. 11a). The compound with the second highest abundance in HL-grown W1 samples was confirmed to be lutonarin (luteolin-6-*C*-glucosyl-7-*O*-glucoside). Similar to saponarin, this flavonoid showed an increased abundance in HL-grown plants from both genotypes. Interestingly, W1 plants grown at HL displayed an eightfold higher amount of lutonarin in comparison to the HL-grown WT (Fig. 11b).

### Localization of saponarin and lutonarin

**Fig. 12:**
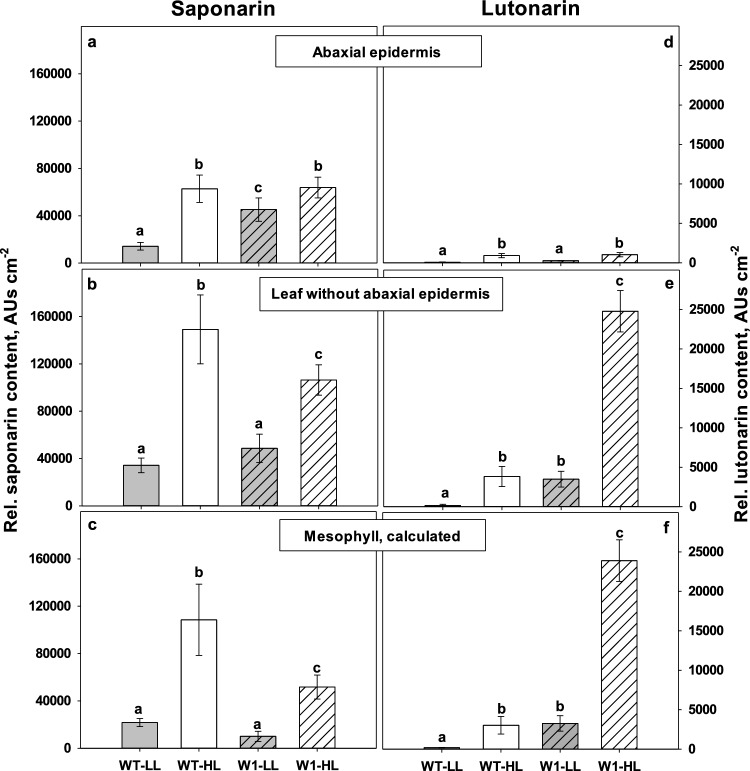
Relative contents of saponarin (**a**–**c**) and lutonarin (**d**–**f**) in the abaxial epidermis, in the segments with the removed epidermis (‘leaf without abaxial epidermis’) and in the mesophyll, respectively, in WT and W1 grown under low (LL, grey bars) and high light (HL, white bars) at 10 das. The data show means ± standard deviation of *n* = 3 samples in total from three independent experiments. Each sample was a pool of 3–5 leaves. The letters indicate statistically different values at a significance level of *P* = 0.05, as determined by two-way ANOVA with genotype and irradiance as factors, followed by pairwise multiple means comparisons by the Holm-Sidak method

To localize the main flavonoids within the leaves, their abaxial epidermis was gently removed from an area of about 1 cm^−2^. With respect to saponarin, the leaf area-related flavonoid content of isolated epidermal peels showed similar trends for the various light conditions and genotypes as the contents of total leaves (Fig. 12, left panels). However, the lutonarin content was strongly reduced in the epidermal peels (Fig. 12, right panels). Since it was not possible to remove the adaxial epidermis at the same time from the remaining piece of mesophyll, the content of this epidermis was extrapolated using measurements of epidermal UV-A transmittance with PAM fluorometry. These measurements revealed that epidermal UV-A transmittance on the adaxial side was very close to that of the abaxial side (Fig. S2). Assuming that not only the total flavonoid content but also the flavonoid composition of both epidermal tissues are similar, the major amount of lutonarin should be located in the mesophyll. Roughly 50% of the saponarin content of WT leaves was expected to be located in the mesophyll, whereas only a minor part of saponarin was estimated for the mesophyll of W1 plants. It is possible that the formation of lutonarin in these leaves occurred to some extent at the expense of saponarin accumulation.

### The ratio of reduced (GSH) to oxidized (GSSG) glutathione

**Fig. 13:**
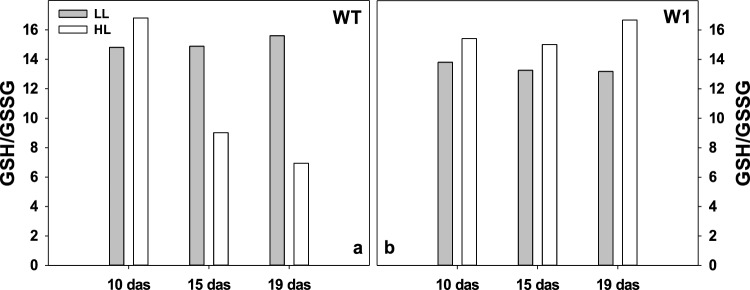
Ratios of reduced (GSH) and oxidized (GSSG) glutathione in WT (**a**) and W1 (**b**) plants grown under low (LL, grey bars) and high light (HL, white bars) on different days after sowing. The data show determinations on pools of 9 primary leaves each, from three independent experiments each comprising three leaves

Glutathione, being a hydrophilic antioxidant found in all cell compartments (Zechmann 2014; Gasperl et al. 2022) may have been involved in ROS protection at HL (Hebbelmann et al. 2012; Heyneke et al. 2013; Dorion et al. 2021). Unlike to LL-grown WT plants, in HL-grown WT the ratio of reduced to oxidized glutathione decreased with increasing age (Fig. 13a).

In contrast, in W1 plants grown either at LL or HL, the ratio of GSH/GSSG did not show any decrease with age but stayed at a similar level as the ratio of 10-day-old WT plants. Rather, the GSH/GSSG ratio of HL-grown W1 showed even slightly higher values than that of LL-grown ones (Fig. 13b).

## Discussion

As reported in previous studies, WHIRLY1-deficient barley plants (W1) are impaired in high-light acclimation at two levels, i.e. photosynthesis and leaf morphology. They have reduced photosynthetic activity in comparison to WT plants, especially when grown under HL, and have a leaf morphology resembling that of shade plants. Chloroplast development and senescence are retarded in these plants (Kucharewicz et al. 2017; Krupinska et al. 2019). Recently, these plants were also shown to be inhibited in photosynthetic acclimation to high irradiance (Saeid Nia et al. 2022). In accordance with the previous results, in this study, the W1 plants showed a reduced electron transport rate (ETR) in comparison to WT plants (Fig. 1). Also, under their growth conditions, they showed typical signs of being exposed to excessive irradiance as indicated by the low epoxidation state (EPS) of the xanthophyll cycle (Fig. 6). A low EPS is indicative of the formation of zea-xanthin as result of a high transthylakoidal proton gradient, activating the enzyme violaxanthin de-epoxidase (Yamamoto 1979; Bilger et al. 1989). It is well known that EPS follows the photochemical quantum yield of PS II under varying illumination (e.g., Bilger and Lesch 1995). Hence, there is no doubt that W1 plants suffered from stress caused by high PFD, especially those grown under HL. It should be noted that the irradiance was always measured parallel to the surface of the barley leaves which were mostly oriented vertically. Horizontal irradiance measured above the plants was 1000 µmol m^−2^ s^−1^, which is comparatively high for a growth chamber experiment. It was expected that the W1 plants, which are impaired in light acclimation, should develop symptoms of photoinhibition when grown under HL conditions, e.g., a reduction in *F*_*V*_/*F*_*M*_ (Aro et al. 1993; Demmig-Adams and Adams 2006; Takahashi and Badger 2011) and eventually loss of chlorophyll (Havaux et al. 2005).

Unexpectedly, the chlorophyll content of W1 plants did increase between 10 and 15 das both under LL and HL conditions indicating that the delayed chloroplast development in W1 plants (Krupinska et al. 2019) was not affected by the light conditions the plants were exposed to. In parallel, *F*_*V*_/*F*_*M*_ starting at a reduced level of about 0.65 at 10 das increased to the WT level at 15 das. Considering that W1 plants cannot enhance photosynthetic capacity at high light (Saeid Nia et al. 2022) as further corroborated by the ETR measurements shown in Fig. 1, they required alternative strategies to cope with excessive light stress. To elucidate these strategies, the fate of the absorbed light was determined. As described above, energy absorbed in PS II is partitioned into three main pathways, Φ(II), Φ(NPQ) and Φ(NO). In leaves of W1 plants the quantum yield of photochemical energy conversion, Φ(II), was reduced. Even at irradiances higher than those encountered during growth in the climate chamber, the W1 plants were able to compensate for the reduced Φ(II) by increased quantum efficiency for non-radiative dissipation, Φ(NPQ) (Fig. 3). Only at the youngest developmental stage and at lower irradiance, the remaining fraction of the energy corresponding to the energy that is passively dissipated by either fluorescence or heat, Φ(NO), was increased in W1 leaves, indicating a stressful situation (Klughammer and Schreiber 2008). However, at 15 das, when chloroplasts were further developed, as indicated by an increased leaf chlorophyll content (Fig. 5), this symptom had disappeared.

To investigate whether oxidative stress affected the ultrastructure of W1 chloroplasts, electron microscopy images were analysed at 10 das. By this approach, it became obvious that plastoglobules were larger and in a higher amount in chloroplasts of HL-grown W1 plants than in those of the WT (Fig. 4). Plastoglobules are plastid lipoprotein particles surrounded by a lipid monolayer and thereby being contiguous with the outer leaflet of thylakoid membranes, which enables an exchange of lipophilic compounds (van Wijk and Kessler 2017). Their multiple functions include the metabolism of prenyl lipids such as tocopherols and the remobilization of thylakoid lipids during stress and senescence. Since a long time it is known that chloroplasts of sun plants have more and larger plastoglobules than chloroplasts of shade plants and that light promotes the accumulation of tocopherols and to a minor extent also xanthophylls in the plastoglobules (reviewed by Lichtenthaler 2013). An increase in the number and/or size of plastoglobules hence is indicative of oxidative stress as a result of excessive light (Bréhélin et al. 2007; Rottet et al. 2015).

To investigate whether W1 plants possess antioxidative mechanisms in addition to non-radiative dissipation to cope with the stressful situation of excessive light, four different types of antioxidative metabolites were measured. Two of them are lipophilic, i.e. zeaxanthin and *α*-tocopherol, and two are mainly hydrophilic, i.e. glutathione and flavonoid glycosides. Based on chlorophyll content, the xanthophyll cycle pool in W1 leaves was very large at higher irradiances, exceeding by far the number of possible xanthophyll binding sites in the light-harvesting complexes (LHCs; Caffarri et al. 2014) (Fig. 6b). The increase of the xanthophyll pool is in line with the strong induction of the expression of *bcHYD,* which codes for the enzyme forming zeaxanthin from *β*-carotene (Sun et al. 1996; Davison et al. 2002). Furthermore, the majority of the xanthophyll cycle pigments were in the de-epoxidized form zeaxanthin (Fig. 6a), supporting the notion that the leaves were suffering from excessive light (Demmig-Adams et al. 1990; Ort 2001). A part of the zeaxanthin pool may have been involved in the mechanism of NPQ, which was strongly enhanced in W1 plants (Fig. 3). In addition to its function in promoting NPQ, zeaxanthin is known to prevent the oxidation of membrane lipids (Havaux and Niyogi 1999; Havaux et al. 2007). Presumably, the larger fraction of zeaxanthin in HL-grown W1 plants was not bound to an LHC but freely located in plastoglobules and in chloroplast membranes. Plastoglobule content was higher in chloroplasts of HL-grown leaves, as reported before (Lichtenthaler 2013). However, Lichtenthaler (2013) also noted that plastoglobules contain only traces of xanthophylls. On the other hand, early light-inducible proteins (ELIPs) which appear only during greening (Montané and Kloppstech 2000) have been reported to bind besides chlorophylls also xanthophylls. As W1 leaves have a delayed development one may speculate that they contained a higher amount of ELIPs, which may also have bound a part of the accumulated zeaxanthin. The exact location of the additional xanthophyll pigments requires further investigations. In a chl *b*-deficient Arabidopsis mutant, free zeaxanthin was shown to be the only carotenoid conferring protection against high-light damage (Havaux et al. 2007). Havaux et al. (2007) suggested that quenching of ^1^O_2_ or scavenging of free radicals by zeaxanthin molecules either located close to the lipid interfaces of LHCII or freely located in the lipid matrix provides photoprotection. Zeaxanthin’s physicochemical interactions with lipids and its orientation in the membrane lipid bilayer (McNulty et al. 2007; Havaux et al. 2007), the extended number of double bonds (Mathews-Roth et al. 1974; Havaux et al. 2007) and its polarity (Wisniewska et al. 2006; Havaux et al. 2007) are considered as important factors determining the specific role of zeaxanthin in protection of thylakoid membrane lipids.

Further analyses of carotenoids revealed that the increase in growth irradiance and excessive excitation energy, as indicated by the large VAZ pool and its low epoxidation state (Fig. 6), was accompanied by a strong enhancement of the lutein content of leaves (Fig. S3). In LHCs, lutein is known as the main quencher of ^3^Chl* (Mozzo et al. 2008; Jahns and Holzwarth 2012; Nezval et al. 2017). Moreover, lutein is involved in the prevention of the formation of ^1^Chl* through its contribution to NPQ (Johnson et al. 2009; Jahns and Holzwarth 2012). However, similar to the xanthophyll cycle pigments, the amount of lutein did by far exceed the number of binding sites in the LHC. Therefore, free lutein may have fulfilled an antioxidative role (Havaux et al. 2007; Demmig-Adams et al. 2020). Havaux et al. (2007) reported that lutein associated with high amounts of zeaxanthin was even more effective in the photoprotection of plants than zeaxanthin itself. In egg yolk liposomal membranes, lutein proved to be an antioxidant as efficient as zeaxanthin (Sujak et al. 1999). Therefore, it is likely that in addition to the free zeaxanthin pool, the elevated amount of lutein was important for the photoprotection of HL-grown W1 plants.

The high zeaxanthin content of W1 leaves at HL correlated positively with the enhanced total pool size of VAZ (Fig. 7). It has been previously hypothesized that the well-known increase of the VAZ pool size at high PFD (Thayer and Björkman 1990; Demmig-Adams et al. 2012) is not regulated by PFD directly, but rather in response to excessive PFD (Bilger et al. 1995; García-Plazaola et al. 2002). As shown in Fig. 7, the VAZ pool size was a close function of EPS, as already observed earlier (Bilger et al. 1995). Furthermore, when EPS increased during further development of the W1 plants, presumably as a response to improved Φ(II) (Fig. 3d), also the VAZ pool size was reduced, keeping the data points close to the previously observed relationship (Fig. 6). This indicates that the VAZ pool size could be regulated by an as yet unknown mechanism that responds to excessive PFD. One factor controlling the VAZ pool size is the expression of the gene encoding *β*-carotene hydroxylase (*HYD,* named also as *CHY* (Kawabata and Takeda 2014)). Both the VAZ pool size and the expression of *HYD*/*CHY1,2* genes in Arabidopsis were shown to increase under HL conditions (Kawabata and Takeda 2014). Using inhibitors of photosynthesis (DCMU, DBMIB), the authors revealed furthermore that the expression of the *CHY* genes, as well as the VAZ pool size, is controlled by the redox state of plastoquinone (even at LL) (Kawabata and Takeda 2014). Overexpression of the *HYD/CHYB* gene in Arabidopsis was shown to enhance tolerance to HL (Davison et al. 2002). The expression of *HYD* was strongly enhanced in the W1 line, especially at high PFD. At the same time, the gene encoding ZEP which catalyses epoxidation of zeaxanthin to violaxanthin (Jahns and Holzwarth 2012), was strongly induced at HL in the WT, but not in the W1 line (Fig. 8b). This pattern of gene expression is in accordance with the increased VAZ pool and the low epoxidation state of the xanthophyll cycle pigments.

In contrast to the zeaxanthin content, the content of tocopherols did neither change in the WT nor in W1 plants in response to HL (Fig. 9). This finding was unexpected considering that *α*-tocopherol has been described as a potent lipophilic antioxidant (Falk and Munné-Bosch 2010; Lushchak and Semchuk 2012), whose level increased together with the level of any ROS species (Kruk et al. 2016) or with increasing irradiance as shown in several studies with Arabidopsis (Lushchak and Semchuk 2012; Collakova and DellaPenna 2003a, b). In contrast to these reports, it has been also observed that the tocopherol content did either not increase with high irradiance (Szymańska et al. 2017) or even declined, e.g. in maize (Leipner et al. 2000; Munné-Bosch and Alegre 2002) and in the cyanobacterial strain *Synechocystis* sp. PCC6803 (Maeda et al. 2005). An increase in tocopherols is regulated at the level of the rate-limiting step of tocopherol biosynthesis which is the transfer of phytyl diphosphate to homogentisate (HGT) catalyzed by the homogentisate phytyltransferase (HPT) enzyme (Collakova and DellaPenna 2003a; Lushchak and Semchuk 2012). Overexpression of *HPT* was shown to increase the tocopherol content of Arabidopsis plants (Collakova and DellaPenna 2003b) and its silencing in tobacco leads to an up to 98% reduction of *α*-tocopherol which was compensated by *γ*-tocopherol. Simultaneous silencing of the *γ*-tocopherol methyltransferase gene (*γTMT*) decreased the total tocopherol level and increased the sensitivity of the plants to various stress conditions imposing oxidative stress dramatically (Abbasi et al. 2007).

In contrast to W1 plants, in older WT plants (19 das) that showed already signs of senescence as declining chlorophyll concentration and RubisCO content (Saeid Nia et al. 2022), the level of *α*-tocopherol increased dramatically (Fig. 9). Indeed, an increased tocopherol content is a characteristic feature of senescence (Falk and Munné -Bosch 2010; Lichtenthaler 2013; Lichtenthaler 1966), which was shown to be delayed in W1 plants (Kucharewicz et al. 2017).

It is known that an interplay between tocopherols and carotenoids is crucial for the prevention of photooxidative stress in Arabidopsis (Kumar et al. 2020). Both lipophilic antioxidants preserve PS II from photoinactivation and protect membrane lipids from photooxidation. Arabidopsis mutants impaired in the biosynthesis of tocopherols (*vte1, vte2*) or zeaxanthin (*npq1, npq4*), respectively, showed no signs of stress when grown in high irradiance. When, however, zeaxanthin formation was inhibited in the *vte1* mutant, PS II was photoinhibited, accompanied by oxidation of lipids and pigments (Havaux et al. 2005). In the barley plants, tocopherols presumably were not required, because the HL treatment led to a strong accumulation of zeaxanthin (Fig. 6). This result is in accordance with the idea that zeaxanthin can compensate for the lack of *α*-tocopherol.

Nevertheless, it was unexpected that the W1 plants accumulate zeaxanthin instead of tocopherols. In a previous study it was shown that thylakoids from W1 plants generate more ROS (H_2_O_2_ and/or superoxide, but not singlet oxygen) than thylakoids from WT plants when illuminated (Swida-Barteczka et al. 2018). Very recently it has been demonstrated that H_2_O_2_ inactivates the enzyme epoxidising zeaxanthin to violaxanthin, i.e. ZEP (Holzmann et al. 2022). In four different dicot species it has been demonstrated that D1 and ZEP during photooxidative stress are degraded coordinately (Bethmann et al. 2019). This degradation might be preceded by the inactivation of ZEP by H_2_O_2_ (Holzmann et al. 2022). Presumably, both, the HL-induced down-regulation of *ZEP* expression (Fig. 8b) and also of its activity, coordinately ensured the retention of a high amount of zeaxanthin under excessive light (Bethmann et al. 2019), which might have been sufficient to prevent accumulation of singlet oxygen enabling the W1 plants to survive this stress.

In addition to zeaxanthin and tocopherols, two hydrophilic antioxidants were compared between WT and W1 plants grown at LL and HL, respectively. Glutathione is the major determinant of the overall cellular redox state (Foyer and Noctor 2005; Mullineaux and Rausch 2005). Any imbalance in the redox situation caused by oxidative stress should shift the ratio between reduced and oxidized glutathione to the side of the oxidized compound (Tausz and Grill 2000; Rahantaniaina et al. 2013; Bloem et al. 2015). In the HL-grown WT leaves, GSH/GSSG declined after 10 das. HL-accelerated premature senescence (Lushchak and Semchuk 2012) in HL-grown WT plants (Kucharewicz et al. 2017; Saeid Nia et al. 2022), as also supported by the increase in *α*-tocopherol, may explain the reduction in the level of the reduced form of glutathione in WT leaves. In contrast, from 10 to 19 das, the GSH/GSSG ratio remained high in the W1 line and was even higher in the HL-grown leaves than in the LL leaves (Fig. 13). Hence, apparently the W1 leaves were able to control oxidative stress to a level not affecting the overall redox state of the cells. Considering that glutathione acts synergistically together with *α*-tocopherol and ascorbate under HL conditions (Kanwischer et al. 2005), the stable GSH/GSSG ratio is in accordance with the unaltered levels of tocopherols in the W1 plants. Correlations among all three members of the ascorbate–glutathione-*α*-tocopherol triad have been observed before (Szarka et al. 2012). That indicates that the content or reduction state of ascorbate, which was not investigated here, might also have not reacted to HL, similar to the other two members of the triad.

Whereas the redox state of the glutathione pool was not specifically altered in the W1 leaves, the composition of the flavonoids changed. In HL-grown leaves, lutonarin increased significantly, and in the W1 leaves to an even higher extent than in WT leaves (Fig. 12). In these leaves, it seemed that the level of lutonarin increased at the expense of the related flavone-glucoside saponarin. While saponarin is a monohydroxyflavone, lutonarin is a dihydroxyflavone. Due to the catechol (ortho-dihydroxylated) group in the latter, these compounds are known to be better antioxidants than the corresponding monohydroxy-compounds (Rice-Evans et al. 1996; Burda and Oleszek 2001; Nezval et al. 2017). Based on this property, the strong increase in the proportion of di- to mono-hydroxy B-ring flavonoids induced by UV radiation (Markham et al. 1998; Tattini et al. 2005; Agati et al. 2009; Agati and Tattini 2010) or, in the absence of UV, by excessive light (Agati et al. 2009; Fini et al. 2011) may be explained as a response enhancing tolerance of oxidative stress. Often, flavonoids are located in the epidermis. In this case when H_2_O_2_ or other oxidative agents would have to diffuse from the origin of oxidative stress, i.e., the photosynthetically active cells, to the epidermis, a function as antioxidant is difficult to imagine. Agati et al. (2002) have shown that in *Phillyrea latifolia* the di-hydroxy-flavonol quercetin is specifically formed in the mesophyll, where it can act as an antioxidant. Moreover, under excessive light stress, quercetin and luteolin glycosides were detected in chloroplasts of *P. latifolia* leaves in association with the chloroplast envelope and were shown to scavenge ^1^O_2_ (Agati et al. 2007), potentially in a complementary action with other singlet oxygen scavengers like carotenoids (Agati et al. 2012).

To investigate the location of lutonarin in the barley leaves, the abaxial epidermis was removed from the leaves. The HPLC analyses revealed that lutonarin was indeed largely absent from the abaxial epidermis (Fig. 12d). Although the lutonarin detected in the remaining part of the leaf could be at least partially located in the adaxial epidermis, which could not be removed simultaneously with the abaxial epidermis from the leaves, it seems tempting to speculate that a significant fraction was present in the mesophyll that contains the chloroplasts (Fig. 12f). It was also shown by Agati and Tattini (2010) that the concentration of the ortho-dihydroxylated flavonoids, quercetin 3-*O*-glycosides and luteolin 7-*O*-glycosides, increased by about 95% in leaves of *Ligustrum vulgare*, where the increase was mainly observed in the mesophyll cells. The finding of the presence of these flavonoids in vacuoles of mesophyll cells and in the chloroplasts close to the ROS generation site in plants was considered as beneficial for the prevention of oxidative damage (Agati and Tattini 2010; Agati et al. 2012; Nezval et al. 2017). By growing under full sunlight and in the absence of UV radiation, these leaves had even a higher concentration of the mentioned flavonoids which accumulated strongly in the palisade parenchyma cells (Agati and Tattini 2010). On the other hand, Kaspar et al. (2010) showed that saponarin, as the main phenolic compound of barley primary leaves, accumulated mainly in the epidermal cells. Recently, it was reported based on the observation of fluorescence induced by Naturstoff reagent A that the epidermal pavement cells of the elite barley cultivar Barke were devoid of flavonoids (Hunt el al. 2021). This observation cannot be supported by our findings in the cultivar Golden Promise, which may be due to either cultivar-specific variations or to the use of different analytical methods. Agati et al. (2002) reported that after staining with Naturstoffreagent A the fluorescence intensity of the standard compound luteolin was ten times higher than that of apigenin. It may be concluded that also the apigenin derivative saponarin has only a low fluorescence emission when stained with this reagent, as was done by Hunt et al. (2021).

To conclude, we demonstrated that the irradiance to which W1 leaves were exposed during growth was excessive for photosynthesis, since the EPS in W1 leaves growing in HL was very low and the light-saturated ETR was reduced in both, LL and HL W1 leaves in comparison to WT. The formation of the flavonoid lutonarin and the strong increase of the VAZ pool size, together with the higher abundance of plastoglobules indirectly indicate that these plants suffered from oxidative stress. However, direct evidence for oxidative stress in the form of an oxidized glutathione pool could not be found, nor was PS II inhibited in leaves at 15 das. Enhanced NPQ may have reduced the light stress, and the carotenoids zeaxanthin and lutein as well as the flavonoid lutonarin likely acted as antioxidants preventing oxidative damage. Since in our study, no changes in the amount of *α*-tocopherol or in the ratio of GSH/GSSG were found in leaves at 10 and 15 das in our study, the *α*-tocopherol-ascorbate–glutathione triad did not appear to play an important role in the protection of the plants. The results of this study indicate that WHIRLY1 coordinates multiple processes enabling plants to respond to high light. In the presence of WHIRLY1, light acclimation at the level of photosynthesis and leaf morphology (Saeid Nia et al. 2022) is prioritized compared with antioxidative defence. In the absence of WHIRLY1, antioxidative mechanisms become more important, whereby it seems that WHIRLY1 also plays a role in the prioritization among the multiple antioxidative defence mechanisms. Although the deficiency of WHIRLY1 compromises barley plants in acclimation to HL (Saeid Nia et al. 2022), photoprotective reactions are still sufficient to prevent serious damage in these plants.

### Author contribution statement

MSN, WB and KK conceived and designed the research. Material preparation, conduction of experiments (except transmission electron microscopy which was done by UR, analysis of flavonoid content and composition which was done by LS, saponarin/lutonarin identification which was done by AGH) were performed by MSN. The glutathione measurements were done with the advice of JS. Graphs and statistical analysis were performed by MSN. The first draft of the manuscript was written by MSN. All authors commented and revised the first version of the manuscript. All authors read and approved the final manuscript.

### Supplementary Information

Below is the link to the electronic supplementary material.

Supplementary file1 (DOCX 204 KB)

## Data Availability

The data that support the findings of this study are available in the Supplementary Information of this article. The raw datasets in this study are available from the first author or corresponding author upon reasonable request.
