# Supplementary material for: How do barley plants with impaired photosynthetic light acclimation survive under high-light stress?
Source: Planta. 2023 Aug 26;258(4):71. doi: 10.1007/s00425-023-04227-8 (PMC10460368; doi:10.1007/s00425-023-04227-8)
Supplement: Supplementary file 1 — Supplementary file1 (DOCX 204 KB) [file 425_2023_4227_MOESM1_ESM.docx]

### Supplementary data

**Tab. S1** A list of genes, their accession numbers and primers, which were used to analyze their relative expression in low and high light

| **Gene** | **Acc. No.** | **5' - 3' forward primer** | **5' - 3' reverse primer** | **bp** |
| --- | --- | --- | --- | --- |
| *HvbcHYD* | HORVU2Hr1G094160 | gggatggcatacatgttcgt | ccatgtggtgtatcttgtgagc | 118 |
| *HvZEP* | HORVU6Hr1G079020 | atgaatgactgccacgttgt | catctgctccaaccaagaga | 106 |
| *HvCHS* | HORVU1Hr1G068790 | gggctcatctccaagaacat | cgctatccaaaagacggagt | 90 |

**Tab. S2** Tocopherol and chlorophyll (chl) content of leaves in WT and W1 plants grown under LL and HL at different days after sowing. Data for chl content was presented before by Saeid Nia et al. (2022). Values are means ± standard deviation of n = 6 samples in total from three independent experiments each comprising 2 leaves(for tocopherol content analysis) and in case of chl content, n = 23–37 leaves in total from three independent experiments each comprising 7–13 leaves (Saeid Nia et al. 2022). The letters indicate statistically different values at a significance level of P = 0.05, as determined by three-way ANOVA, followed by pairwise multiple means comparisons by the Holm-Sidak method

| **Samples name** | **das** | **α-tocopherol (µg cm^-2^)** | **β-tocopherol (µg cm^-2^)** | **γ-tocopherol (µg cm^-2^)** | **Total-tocopherol (µg cm^-2^)** | **Chl *a*+*b* (nmol cm^-2^)** |
| --- | --- | --- | --- | --- | --- | --- |
| **WT-LL** | 10 | 0.30±0.04^a^ | 0.002±0.0006^a^ | 0.008±0.001^a^ | 0.30±0.04^a^ | 41.2±5.2^a^ |
|  | 15 | 0.40±0.11^ab^ | 0.002±0.0008^a^ | 0.007±0.002^a^ | 0.40±0.12^a^ | 45.4±3.5^a^ |
|  | 19 | 0.63±0.64^b^ | 0.144±0.01^b^ | 0.008±0.002^a^ | 0.79±0.07^b^ | 45.0±4.7^a^ |
| **WT-HL** | 10 | 0.35±0.07^a^ | 0.007±0.003^a^ | 0.01±0.002^a^ | 0.40±0-071^ab^ | 44.6±5.3^a^ |
|  | 15 | 0.48±0.15^ab^ | 0.003±0.001^a^ | 0.009±0.005^a^ | 0.5±0.15^ab^ | 42.6±7.9^a^ |
|  | 19 | 2.21±0.31^c^ | 0.275±0.073^c^ | 0.12±0.05^b^ | 2.61±0.39^d^ | 32.9±9.2^b^ |
| **W1-LL** | 10 | 0.28±0.06^a^ | 0.002±0.001^a^ | 0.01±0.004^a^ | 0.29±0.06^a^ | 28.7±3.8^c^ |
|  | 15 | 0.41±0.13^ab^ | 0.004±0.002^a^ | 0.008±0.004^a^ | 0.42±0.14^ab^ | 36.5±5.5^d^ |
|  | 19 | 0.44±0.08^ab^ | 0.16±0.02^b^ | 0.011±0.005^a^ | 0.61±0.1^ab^ | 39.2±4.0^d^ |
| **W1-HL** | 10 | 0.28±0.03^a^ | 0.007±0.009^a^ | 0.01±0.004^a^ | 0.29±0.04^a^ | 16.7±2.9^b^ |
|  | 15 | 0.27±0.07^a^ | 0.004±0.003^a^ | 0.01±0.006^a^ | 0.28±0.074^a^ | 30.9±5.9^e^ |
|  | 19 | 0.41±0.13^ab^ | 0.212±0.073^bc^ | 0.012±0.004^a^ | 0.64±0.20^ab^ | 30.7±9.7^eb^ |

**
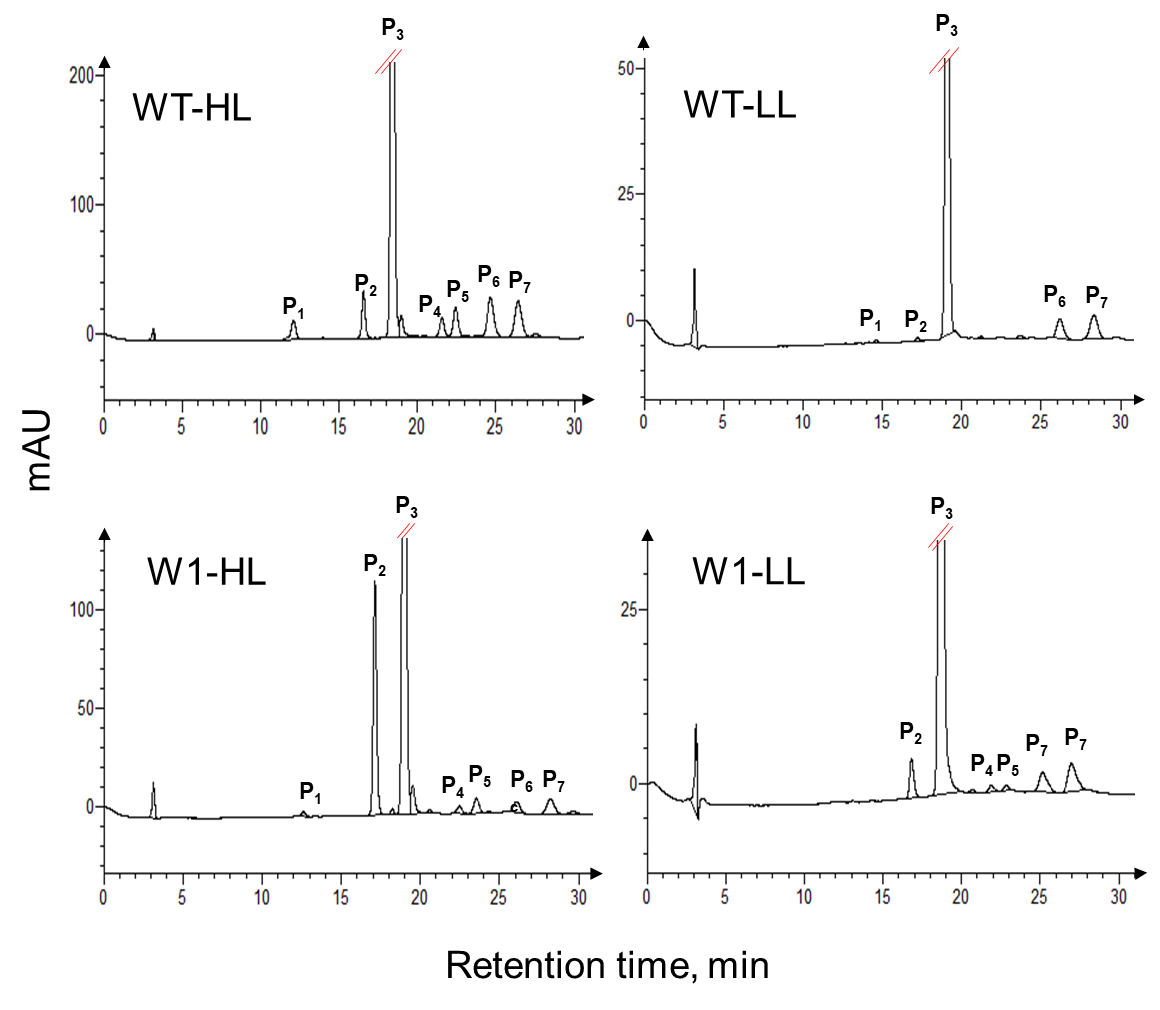
**

**Fig. S1** Representative HPLC chromatograms for W1 and WT grown under low and high light detected at 313 nm. For more details about the peaks see Tab. S3. Please note, that the scale of the y-axis differs between the different chromatograms.

**Tab. S3** List of compounds identified in the chromatograms (see Fig. S1). References are abbreviated as follows: [a] = (Nezval et al., 2017); [b] = (Kolb and Pfündel, 2005); [c] = (Ferreres et al., 2008); [d] = (Brauch et al., 2018). UV-VIS ± 2 nm, retention time ± 0.5 min. Sh stands for "shoulder" and describes a distinct elevation within a band, but not corresponding to the absorption maximum of the band**.**

| **ID** | **Name** | **UV-VIS (ג_max_ nm)** | **Retention time (min)** | **References** |
| --- | --- | --- | --- | --- |
| P1 | Feruloylquinic acid | 243, 326 | 12.66 | [a], [b], [c], [d] |
| P2 | Lutonarin | 269, 350 | 17.19 | [a], [b], [c], [d] |
| P3 | Saponarin | 270, 336 | 19.03 | [a], [b], [c], [d] |
| P4 | Luteolin derivative 1 | 270, 343 | 22.55 | [a], [b] |
| P5 | Luteolin derivative 2 | 254sh, 271, 340 | 23.63 | [a], [b] |
| P6 | Apigenin derivative 1 | 271, 336 | 26.18 | [a], [b] |
| P7 | Apigenin derivative 2 | 271, 331 | 28.35 | [a], [b] |

Brauch D, Porzel A, Schumann E, Pillen K, Mock H-P (2018) Changes in isovitexin-O-glycosylation during the development of young barley plants. Phytochemistry 148:11-20 <https://doi.org/10.1016/j.phytochem.2018.01.001>

Ferreres F, Andrade PB, Valentão P, Gil-Izquierdo A (2008). Further knowledge on barley (*Hordeum* *vulgare* L.) leaves O-glycosyl-C-glycosyl flavones by liquid chromatography-UV diode-array detection-electrospray ionisation mass spectrometry. J Chrom A 1182:56-64

Kolb CA, Pfündel EE (2005) Origins of non-linear and dissimilar relationships between epidermal UV absorbance and UV absorbance of extracted phenolics in leaves of grapevine and barley. Plant Cell Environ 28:580-590

Nezval J, Štroch M, Materová Z, Špunda V, Kalina J (2017) Phenolic compounds and carotenoids during acclimation of spring barley and its mutant *Chlorina f2* from high to low irradiance. Biol Plant 61:73–84. https://doi.org/10.1007/s10535-016-0689-0

**Fig. S2** The UV-A absorbance measured in the adaxial epidermis as a function of UV-A absorbance in the abaxial epidermis in WT and W1 plants grown under different irradiances at day 10 after sowing. The regression equations are y=0.8863x + 0.0147 (r^2^=0.863) for WT and y=08549x-0.0285 (r^2^=0.781) for W1

**Fig. S3** Leaf lutein content per chlorophyll *a*+*b* as a function of incident irradiance (PFD) (a) and as a function of EPS (b) in WT and W1. Circles and diamonds denote WT and W1 plants, respectively. Open and filled symbols denote 10 and 15 day old plants, respectively. Data are taken from two experiments, one in which sampling was only on das 10, and another one in which sampling was on das 10 and 15
